# An Attenuated Zika Virus Encoding Non-Glycosylated Envelope (E) and Non-Structural Protein 1 (NS1) Confers Complete Protection against Lethal Challenge in a Mouse Model

**DOI:** 10.3390/vaccines7030112

**Published:** 2019-09-12

**Authors:** Arun S. Annamalai, Aryamav Pattnaik, Bikash R. Sahoo, Zack P. Guinn, Brianna L. Bullard, Eric A. Weaver, David Steffen, Sathish Kumar Natarajan, Thomas M. Petro, Asit K. Pattnaik

**Affiliations:** 1 School of Veterinary Medicine and Biomedical Sciences, University of Nebraska-Lincoln, Lincoln, NE 68583, USA; tiger05009@gmail.com (A.S.A.); aryamavpattnaik@ymail.com (A.P.); bsahoo@huskers.unl.edu (B.R.S.); dsteffen1@unl.edu (D.S.); 2 Nebraska Center for Virology, University of Nebraska-Lincoln, Lincoln, NE 68583, USA; bbullard@huskers.unl.edu (B.L.B.); eweaver2@unl.edu (E.A.W.); snatarajan2@unl.edu (S.K.N.); tpetro@unmc.edu (T.M.P.); 3 Department of Oral Biology, University of Nebraska Medical Center, Lincoln, NE 68583, USA; zack.guinn@huskers.unl.edu; 4 School of Biological Sciences, University of Nebraska-Lincoln, Lincoln, NE 68583, USA; 5 Department of Nutrition and Health Sciences, University of Nebraska-Lincoln, Lincoln, NE 68583, USA

**Keywords:** Zika virus, NS1 protein, glycosylation, attenuation, vaccine candidate

## Abstract

Zika virus (ZIKV), a mosquito-transmitted flavivirus, emerged in the last decade causing serious human diseases, including congenital microcephaly in newborns and Guillain-Barré syndrome in adults. Although many vaccine platforms are at various stages of development, no licensed vaccines are currently available. Previously, we described a mutant MR766 ZIKV (m2MR) bearing an E protein mutation (N154A) that prevented its glycosylation, resulting in attenuation and defective neuroinvasion. To further attenuate m2MR for its potential use as a live viral vaccine, we incorporated additional mutations into m2MR by substituting the asparagine residues in the glycosylation sites (N130 and N207) of NS1 with alanine residues. Examination of pathogenic properties revealed that the virus (m5MR) carrying mutations in E (N154A) and NS1 (N130A and N207A) was fully attenuated with no disease signs in infected mice, inducing high levels of humoral and cell-mediated immune responses, and protecting mice from subsequent lethal virus challenge. Furthermore, passive transfer of sera from m5MR-infected mice into naïve animals resulted in complete protection from lethal challenge. The immune sera from m5MR-infected animals neutralized both African and Asian lineage viruses equally well, suggesting that m5MR virus could be developed as a potentially broad live virus vaccine candidate.

## 1. Introduction

Zika virus (ZIKV) belongs to the family *Flaviviridae* and genus Flavivirus. It is primarily transmitted by *Aedes* species of mosquito, but vertical and sexual transmissions have also been reported [1,2,3]. Historically, the majority of ZIKV infections in humans were asymptomatic, but in about 20% of cases, were associated with mild flu-like illnesses with disease symptoms, such as fever, conjunctivitis, rash, and joint pain, which may last less than a week [2]. However, the virus remerged in the last decade in the Americas, the Caribbean, and the Pacific regions [4], causing severe diseases. Since its introduction into the Americas in 2014, over one million individuals are suspected to have been infected with ZIKV [5]. So far, 27 countries and territories in the Americas have reported the association between the ZIKV infections and Congenital Zika Syndrome, Guillain-Barré syndrome (GBS), and other neurological disorders [6,7,8].

ZIKV is enveloped and consists of approximately 11 kb positive-sense, single-stranded RNA genome. The viral genome has a 5′ cap and 5′- and 3′-UTRs and contains a single open reading frame (ORF), which is translated as a single polyprotein at the endoplasmic reticulum (ER) membrane [9,10]. The polyprotein is cleaved and processed by viral and host proteases to generate three amino-terminal structural proteins (capsid, C; pre-membrane, prM; envelope, E) and seven carboxyl-terminal non-structural (NS) proteins (NS1, NS2A, NS2B, NS3, NS4A, NS4B, and NS5) [10]. The structural proteins are involved in virion structure, assembly, attachment, and entry into host cells [11]. The NS proteins are associated with the ER and participate in essential functions, such as genome replication, virion assembly, polyprotein processing, and evasion of host antiviral responses [12,13,14]. Importantly, it appears that the flavivirus E protein elicits neutralizing antibody responses [15,16,17], while the NS1 protein induces non-neutralizing antibodies [18]. However, these NS1 antibodies have been shown to mediate constant region (Fc)-dependent cell-mediated immunity against ZIKV [4,19,20,21,22,23]. 

In dengue virus (DENV) and other flavivirus infected cells, NS1 is synthesized as a soluble monomer, which rapidly dimerizes in the lumen of ER [24,25,26]. NS1 exists as various oligomeric forms in different cellular locations [27,28,29]. Dimeric NS1 is localized in the lumen of the ER, secretory vesicles, or on the cell surface (membrane-bound NS1, mNS1) [29]. Intracellular NS1 plays an essential role in virus replication as a cofactor and has been shown to co-localize with dsRNA intermediate in replication complexes [30,31]. A proportion of NS1 dimers form hexamers in the Golgi that are transported to the cell surface and secreted (secreted NS1, sNS1) [32]. NS1 also contains epitopes for protective antibodies [4,33]. NS1 is a multifunctional, highly immunogenic protein and plays a role in pathogenesis and immune evasive functions [34,35]. However, the role of NS1 in immunopathogenesis and the potential of NS1-mediated immune responses in protection against ZIKV remain incompletely understood. 

Sequence analysis and experimental studies have revealed that structural and non-structural proteins of flaviviruses contain several N-linked glycosylation sites [32,36,37,38]. Particularly, it has been shown that the glycosylation sites in E and NS1 proteins are conserved among members of the flavivirus genus [39,40,41,42]. In majority of isolated ZIKV strains, the E protein is glycosylated at asparagine 154 (N154) located in the domain I (DI) [43], whereas there are two glycosylation sites present in NS1 protein: one at N130 located in the wing domain and the other at N207 located in the continuous β-ladder domain [44]. Although, most flavivirus NS1 contain N-linked glycosylation sites at N130 and N207, in West Nile virus (WNV), an additional glycosylation site is present at position N175, while the second glycosylation site in yellow fever virus (YFV) is located at N208 [36,40,41,42].

The function of NS1 glycosylation has been studied for other flaviviruses. Mutation of the N207 site in DENV-2 led to reduced virus growth in cell culture and decreased neurovirulence in mice [45]. On the other hand, mutation at N130 of chimeric tick-borne encephalitis (TBE)/DEN-4 virus [containing membrane and envelope (ME ) structural genes of TBE virus (TBEV) and remaining genes from (DENV-4)] resulted in reduced virus titers in cell culture and decreased mouse neurovirulence as opposed to increased neurovirulence in an N207 mutant [46,47]. In YFV, ablation of the N130 site alone or in combination with N207 resulted in reduced virus yields, cytopathic effect, NS1 secretion, plaque size, and neurovirulence [48]. In WNV, ablation of NS1 glycosylation sites resulted in attenuation of neuroinvasive properties of the virus, while the removal of glycosylation sites in both E and NS1 proteins completely attenuated virus neuroinvasiveness and neurovirulence in a mouse model [42]. Overall, these studies suggest that NS1 glycosylation appears to play a vital role in viral replication and pathogenesis. 

We previously generated E glycosylation mutant ZIKVs, in which four amino acids spanning the glycosylation site were deleted (m1MR) or N154 was changed to A154 (m2MR) and showed that the mutant viruses were significantly attenuated in pathogenicity and defective in neuroinvasion [49]. In the genomic background of the m2MR mutant virus, we explored whether additional incorporation of mutations in the NS1 glycosylation sites would further attenuate the resulting virus to be used as a potential live virus vaccine candidate. Our data presented here showed that the mutant ZIKV that encoded non-glycosylated E and NS1 proteins was highly attenuated in pathogenicity and conferred complete protection of animals upon lethal challenge with ZIKV.

## 2. Materials and Methods 

### 2.1. Ethics Statement 

This study was performed per the recommendations in the Guide for the Care and Use of Laboratory Animals of the National Institutes of Health. The protocol (# 1323) was approved by the Institutional Animal Care and Use Committee (IACUC) at the University of Nebraska-Lincoln (UNL). All animals were housed in the Life Sciences Annex building at the university. All procedures were conducted under anesthesia with isoflurane, and all efforts were made to minimize animal suffering.

### 2.2. Cells and Viruses 

Vero, the African green monkey kidney cells (*Cercopithecus aethiops*, CCL-81) and C6/36 mosquito cells (*Aedes albopictus*, CRL-1660) were obtained from ATCC (Manassas, VA, USA). Vero cells were grown and maintained in Dulbecco’s modified Eagle medium (DMEM) (Invitrogen, Carlsbad, CA, USA) supplemented with 10% fetal bovine serum (heat-inactivated) (FBS; HyClone Laboratories, Logan, UT, USA), 1% penicillin/streptomycin (PS) (Invitrogen, Carlsbad, CA, USA) in humidified chamber with 5% CO_2_ at 37 °C, whereas C6/36 cells were maintained similarly at 32 °C. African lineage parental ZIKV strain MR766 and the infectious clone derived recombinant MR766 (rMR) virus have been described previously [49]. American/Asian lineage PRVABC59 ZIKV virus was obtained from Barbara Johnson at the Centers for Disease Control and Prevention, Fort Collins, CO, USA. American/Asian lineage MEX1–7 strain of ZIKV was obtained from S. Byrareddy (University of Nebraska Medical College). Virus stocks were prepared by infecting a confluent monolayer of Vero cells at multiplicity of infection (MOI) of 0.1 plaque-forming unit (PFU) per cell and incubating in virus growth medium (VGM) (DMEM containing 2% FBS, 20 mM hydroxyethyl piperazine ethane sulfonic acid (HEPES), 1 mM sodium pyruvate, non-essential amino acids, and 1% PS) for four days, as described previously [49,50]. Multistep growth analysis was conducted using the MOI of 0.1 PFU/cell. Following adsorption of the virus, the inoculum was removed, cells were washed twice in phosphate-buffered saline (PBS), VGM was added, and the cells were incubated at 37 °C/5% CO_2_ incubator. Small aliquots of infected cell culture supernatants (CCS) at various days post-infection (dpi) were collected, clarified, and stored at −80 °C before virus titration. For glycan analysis of NS1 protein, cells were infected at an MOI of 1 PFU/cell, and CCSs were collected at 2 dpi for further analysis.

### 2.3. Reagents and Antibodies

Restriction enzymes, DNA modifying enzymes, ProtoScript II First Strand cDNA Synthesis Kit, Q5 High Fidelity PCR Kit, endoglycosidase H (Endo H), and peptide-N-glycosidase F (PNGase F) were obtained from New England Biolabs (NEB) (Ipswich, MA, USA). Superscript II was obtained from Invitrogen (Carlsbad, CA, USA). TransIT-mRNA transfection reagent was obtained from Mirus Bio (Madison, WI, USA). Oligonucleotide primers and probes for DNA amplification and qRT-PCR were obtained from Sigma (St. Louis, MO, USA) and IDT (Coralville, IA, USA). mMESSAGE mMACHINE T7 ULTRA Transcription Kit was from Ambion (Austin, TX, USA). Anti-flavivirus monoclonal antibody D1-4G2-4-15 that reacts with ZIKV E protein was obtained from EMD Millipore (Billerica, MA, USA). Rabbit polyclonal anti-NS1 antibody was obtained from GeneTex (Irvine, CA, USA). Mouse monoclonal antibody to GAPDH was obtained from Santa Cruz Biotechnology (Dallas, TX, USA). Secondary antibodies were obtained from Sigma (St. Louis, MO, USA) and Invitrogen (Carlsbad, CA, USA).

### 2.4. qRT-PCR and Plaque Assay

Virus genome copy numbers in various tissue samples were determined by quantitative reverse transcription and polymerase chain reaction (qRT-PCR), and infectious virus by plaque assay on monolayers of Vero cells, as described previously [49].

### 2.5. Generation of Mutant Viruses 

Mutant viruses were generated by site-directed mutagenesis of the previously described infectious clone developed in our laboratory [49] using the primers described in Table 1. The asparagine residues at 130 and 207 of the NS1 protein were replaced with alanine residues either separately or in combination (N130A, N207A, N130A, and N207A) in the background of m2MR virus [49], which lack E protein glycosylation site (N154A). N130A mutant was generated with the use of the mutagenic primer 2 and the two outer primers 1 and 5; N207A mutant was generated using the mutagenic primer 3 and the two outer primers 1 and 5; N130A/N207A double mutant was generated using the mutagenic primers 2 and 4 and the outer primers 1 and 5. PCR amplified products were cloned into a full-length infectious cDNA clone. Mutations were confirmed by sequencing of the clone. In vitro RNA transcripts were synthesized from mutant cDNA clones using T7 RNA polymerase, as previously described [49]. Vero cells were transfected with RNA (~2 μg) using TransIT^®^-mRNA transfection kit, as per the manufacturer’s recommendations. At five days post-transfection (dpt), immunofluorescence was performed using 4G2 antibody targeting the E protein of ZIKV, as previously described [49]. For virus recovery, CCSs were collected at 5–7 dpt, and the infectious virus yield was determined by plaque assay on Vero cells.

### 2.6. Mouse Experiments

Three- to four-week-old A129 mice (*Ifnar1*^−/−^) were obtained from the Jackson Laboratory (Bar Harbor, ME, USA). Initially, the animals were acclimatized for 4–5 days. The mice were injected subcutaneously (s.c.) with PBS or 1000 PFU of rMR, or the mutant (m2MR and m5MR) viruses diluted in 100 μL of PBS. For challenge studies, all the mice were injected s.c. with 10,000 PFU of rMR on day 28 post-infection. For passive transfer experiment, the mice were injected s.c. with 200 μL of heat-inactivated (56 °C for 30 min) m5MR virus-infected mouse serum or naïve mouse serum as control. Two hours post-transfer (hpt), mice were injected s.c. with 1000 PFU rMR virus and were monitored for disease signs twice daily. Change in body weight, disease signs, and survival was recorded daily. We assigned scores for disease signs, as described previously [49]. Briefly, clinical symptoms were scored as 0, Normal; 1, ruffled fur; 2, conjunctivitis, lethargy, hunched posture; 3, one hind limb paralysis; 4, both hind limb paralysis; 5, all four limb paralysis; 6, moribund and euthanization; 7, dead. Mice were considered as moribund when they appeared very lethargic, lost more than 20% body weight, or showed limb paralysis and were euthanized. Blood was collected by retro-orbital bleeding under anesthesia. Mice were euthanized by CO_2_ inhalation, and tissue samples were collected. To assess the viral load in blood and tissues, we performed qRT-PCR for genome copies and plaque assay for infectious viral titer. Tissues were examined for pathological changes by histopathology. The qRT-PCR and histopathological evaluation were performed, as described previously [49].

### 2.7. Plaque Reduction Neutralization Test

Plaque reduction neutralization test (PRNT) to measure ZIKV neutralizing antibody titers was performed. Vero cells were plated in 12 well plates 24 h before infection to have confluency of 90–100% at the time of infection. All serum samples were heat-inactivated at 56 °C for 30 min prior testing. The serum was diluted 1/100 followed by serial two-fold dilutions up to 1/12,800. Naïve mouse serum served as the negative control in these studies. An equal volume of virus suspension containing ~200 PFU was mixed with diluted serum samples and incubated at 37 °C for 1 h. After incubation, the mixture was used to infect the cells in the plates at 37 °C for 1 h with rocking every 10 min. Following infection, the inoculum was removed, and the cells were overlaid with medium containing 1% low gelling temperature (LGT) agarose in VGM as described for plaque assay. After incubation for 5 days at 37 °C, the plaques were counted manually. Antibody titers were determined as the reciprocal of the serum dilution that inhibited 50% of the virus infectivity (PRNT_50_), and geometric mean titers (GMT) were calculated from the PRNT_50_ values.

### 2.8. Western Blotting

For Western blotting, 8% polyacrylamide gel containing sodium dodecyl sulfate (SDS-PAGE) was used. After electrophoresis, the proteins were transferred to polyvinylidene difluoride (PVDF) membrane using a semi-dry transfer system (Bio-Rad, Hercules, CA, USA). The membrane was blocked with blocking buffer (5% skim milk in tris-buffered saline (TBS) and 0.05% Tween 20) for 1–2 h. After blocking the membrane, the specific primary antibody (anti-NS1 protein at 1:1000 dilution) was added and incubated overnight at 4 °C. The membrane was washed with TBS buffer containing 0.05% Tween 20 three times, 5–10 min each. Secondary antibody (Goat Anti-Rabbit HRP conjugated) at 1:5000 dilution was added and incubated for 1–2 h at room temperature. The membrane was washed with the TBS buffer. Finally, the membrane was treated with Pierce™ ECL Western Blotting Substrate (Thermo Fisher Scientific, Waltham, MA, USA) for 5 min and exposed to X-ray film or ChemiDoc Imaging System (Bio-Rad, Hercules, CA, USA) for the required time to visualize the bands.

### 2.9. Acetone Precipitation of Proteins

For glycan analysis of NS1 proteins in the supernatants, Vero cells were infected with the recombinant wt and mutant viruses at an MOI of 1 PFU/cell. The CCSs were collected at 2 dpi, and proteins were concentrated by acetone precipitation. Briefly, one volume of CCS was mixed with four-volume of cold acetone (−20 °C) in a microcentrifuge tube and incubated for 60 min at −20 °C. The precipitated proteins were pelleted by centrifugation at 4 °C for 10 min at 13,000–15,000× *g*. The supernatant was discarded, and the pellet was air-dried at RT for 30 min. The pellet was resuspended in 1X RIPA buffer with freshly added protease inhibitor cocktail (2 mM PMSF, 1 mM Leupeptin). The proteins were digested with PNGase F as per manufacturer’s recommendations. 

### 2.10. Antibody Isotyping by ELISA 

ELISA plates were coated with ZIKV envelope protein (#596088, MyBiosource, Inc., San Diego, CA, USA) at 2 μg/mL in PBS for 16 h at 4 °C. After addition in blocking buffer (Invitrogen, Superblock, Thermo Fisher Scientific, Waltham, MA, USA serum samples diluted in blocking buffer (1:50) were added and incubated at room temperature for 2 h. After removal of excess serum, biotinylated goat anti-mouse IgG, biotinylated rat anti-mouse IgG1, or biotinylated rat anti-mouse IgG2a (Biolegend, San Diego, CA, USA; catalog #405303, #406603, and #407103, respectively) were added in blocking buffer at 1 μg/mL and incubated for 1 h. After three washes with PBS, streptavidin horseradish peroxidase was added (1:1000; BD-Pharmingen, San Diego, CA, USA). After 30 min of incubation and three washes with PBS, 3,3-,5,5-tetramethylbenzidine-hydrogen peroxide solution was added to each well. IgG subclass specific anti-ZIKV envelope protein was measured by determining optical densities at the 450-nm wavelength (OD450) with a reference OD570 using an ELISA plate reader.

### 2.11. ELISPOT Assay

The ZIKV T-cell epitopes were previously mapped, and the assay was performed, as described [51]. An overlapping peptide array of the ZIKV strain PRVABC59 E protein from BEI Resources (Catalog No. NR-50553) was used. Peptides 1 and 2 (Table 2) were used for CD8+-specific response, whereas the rest of the peptides were used for CD4+-specific response, as reported previously [51]. Ninety-six well polyvinylidene difluoride-backed plates (MultiScreen-IP, Millipore, Burlington, MA, USA) were coated with 50 μL of anti-mouse IFN-γ mAb AN18 (5 μg/mL; Mabtech, Cincinnati, OH, USA) overnight at 4 °C. Plates were washed and blocked with RPMI at 37 °C for 1 h. Splenocytes were isolated from mice using a 40 μm Nylon cell strainer (BD Labware, Franklin Lakes, NJ, USA), red blood cells were lysed using ammonium chloride-potassium (ACK) lysis buffer, and the splenocytes were resuspended in complete RPMI at a concentration of 4 × 10^6^ cells/mL. The CD8 and CD4 peptide dilutions contained each peptide at a concentration of 5 μg/mL. Equal volumes (50 μL) of the single-cell suspension splenocytes and peptide dilutions were added to the wells in duplicate. Plates were incubated overnight at 37 °C with 5% CO_2_, washed 6X with PBS, and incubated with 100 μL of biotinylated anti-mouse IFN-γ mAb (1:1000 dilution; Mabtech) diluted in PBS with 1% FBS for 1 h at room temperature. Following washing 6X with PBS, the plates were incubated with 100 μL of streptavidin-alkaline phosphatase conjugate (1:1000 dilution; Mabtech) diluted in PBS containing 1% FBS. After 1 h at room temperature, the plates were washed 6x with PBS. To develop, 100 μL of 5-bromo-4-chloro-3’-indolyphosphate/nitro-blue tetrazolium (BCIP/NBT) (Plus) alkaline phosphatase substrate (Thermo Fisher Scientific, Waltham, MA, USA) was added to each well, and development was stopped by washing several times in distilled water. The plates were air-dried, and spots were counted using an automated ELISPOT plate reader (AID iSpot Reader Spectrum, Strassberg, France). Results are expressed as spot-forming cells (SFC) per 10^6^ splenocytes.

### 2.12. Statistical Analysis

Data were analyzed using GraphPad Prism software version 6.0. Unpaired two-tailed Student’s *t*-test, Mann–Whitney test, or Kruskal–Wallis test was performed for pairwise comparisons between the groups to determine significant differences in viral loads (RNA levels, infectious titer), clinical score, PRNT_50_, ELISA, ELISPOT. Data were represented as either mean ± SEM or mean ± SD.

## 3. Results

### 3.1. Recovery and Characterization of E/NS1 Glycosylation Mutant Viruses

**Figure 1:**
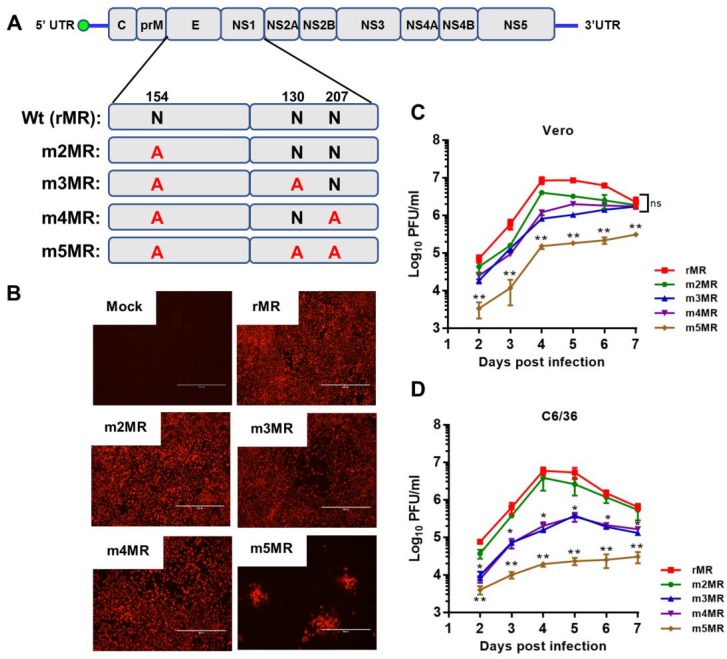
Characterization of the envelope (E)/non-structural protein 1 (NS1) glycosylation mutant viruses. (**A**) The ZIKV (Zika virus) genome and the encoded polyprotein, showing glycosylation sites in E (N154) and NS1 (N130 and N207). The asparagine residue at each of these sites was mutated to an alanine residue. (**B**) Two micrograms of in vitro transcribed RNA, from each of the mutant clones or the wt(rMR) clone, were transfected into Vero cells in 6-well plates. After 5 days, the transfected cells were processed for immunofluorescence staining using 4G2 monoclonal antibody to detect the E protein. Scale bar: 400 μm. Multistep growth of rMR, m2MR, m3MR, m4MR, and m5MR viruses in Vero (**C**) and C6/36 (**D**) cells. The data show mean values with error bars representing standard deviation from three independent experiments. Mann–Whitney test and Kruskal–Wallis test were used to determine significance between m2MR and other mutant viruses at different times post-infection. **, *p* < 0.01; *, *p* < 0.05; ns, non-significant.

Our previous studies showed that mutant MR766 ZIKVs encoding non-glycosylated E protein was highly attenuated in an immunodeficient mouse model [49]. Although the mutant virus-infected animals showed no disease signs during the first six days, a small percentage (less than 10%) of the animals exhibited mild signs of sickness, including weight loss and hind limb paralysis at 9 dpi, but recovered completely without any further disease signs for up to 2 months. We hypothesized that incorporation of glycosylation mutation in the NS1 in the background of E mutant virus would result in more complete attenuation. Therefore, we generated three additional mutant viruses in the background of the m2MR mutant virus, which encodes a non-glycosylated E protein, to study the effect of NS1 glycosylation in virus replication and pathogenesis. The asparagine residues in the glycosylation sites in NS1 (N130 and N207) were changed to alanine residues in the genomic background of the E glycosylation mutant virus (m2MR), and the mutant viruses (Figure 1A) (m3MR, with mutations N154A in E and N130A in NS1; m4MR, with mutations N154A in E and N207A in NS1; m5MR with mutations N154A in E, N130A and N207A in NS1) were recovered from the mutant infectious clones, as described previously [49]. Interestingly and unlike the cells transfected with transcripts from m2MR, m3MR, and m4MR clones, the cells transfected with RNA from m5MR clone showed localized foci of fluorescence 6 days after transfection (Figure 1B), indicating a very restrictive growth of this virus. Nevertheless, the m5MR virus could be readily recovered from the transfected cells. Multistep growth kinetics studies revealed that the growth of single substitution NS1 mutant viruses (m3MR and m4MR) was lower at 4 dpi in Vero cells, but they eventually grew to similar titers compared to m2MR virus by 6 to 7 dpi (Figure 1C). The double substitution NS1 mutant virus (m5MR), on the other hand, grew to significantly lower titers at all-time points examined. In C6/36 mosquito cell line, both m3MR and m4MR viruses grew to lower titers, while the m5MR virus grew to significantly lower titers at all times examined compared to m2MR virus (Figure 1D). These results suggest that glycosylation of NS1 is more important for the efficient growth of virus in mosquito cells than in Vero cells. Sequencing of the E and NS1 coding region of the mutant viruses showed that all the mutant viruses retained the engineered mutations after six passages in Vero cells, and no other mutations/deletions in the E and NS1 protein-coding regions were observed.

### 3.2. Glycosylation Status and Secretion of NS1 Protein from Mutant Virus-Infected Cells

**Figure 2:**
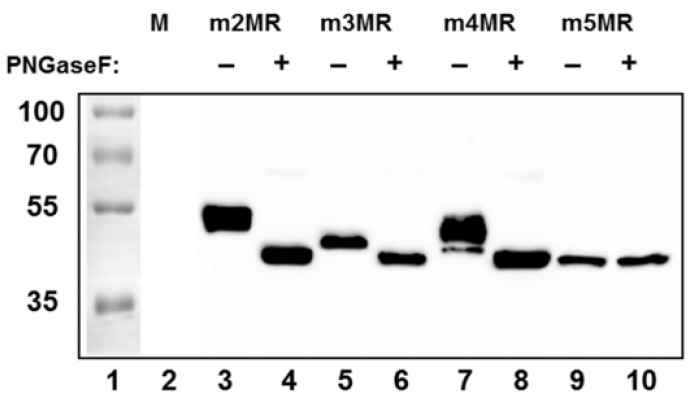
Glycosylation status and secretion of NS1 protein from the mutant virus-infected cells. Cells in 6-well plates were mock-infected (M) or infected with the mutant viruses (shown on top) at an MOI (multiplicity of infection) of 1. Cell culture supernatants were collected at 48 dpi, and proteins from an equal volume of supernatants were concentrated by acetone precipitation, digested with PNGase F (+), or left undigested (−) and analyzed by Western blotting using anti-NS1 antibody. Relative migration of molecular mass markers (in kDa) is shown on the left.

Since NS1 is secreted from cells infected with wild-type ZIKV, we examined the status of glycosylation of the NS1 protein and whether it is secreted from the mutant virus-infected cells. To address this, we infected Vero cells at an MOI of 1, and equal amounts of cell culture supernatants collected at 48 dpi were concentrated by acetone precipitation and either left undigested or digested with PNGase F (an enzyme that removes the glycan moiety from protein backbone) before being analyzed by Western blotting using an anti-NS1 antibody. The NS1 protein from m2MR virus-infected cell supernatant, which has the two intact glycosylation sites, upon digestion, migrated faster (Figure 2, lane 4) than the undigested NS1 (lane 3). The NS1 from m3MR- or m4MR-infected supernatants, each of which has a single glycosylation site, migrated faster (Figure 2, lanes 5 and 7) than the undigested protein from the m2MR (lane 3) but slower than the corresponding digested proteins (lanes 6 and 8). On the other hand, no difference in migration of NS1 in undigested or digested m5MR-infected cell supernatant was observed (Figure 2, lanes 9 and 10), indicating that the NS1 protein produced from this virus-infected cells lacked PNGase F sensitive glycans. Although m3MR and m4MR viruses possessed single glycosylation site mutations, the NS1 proteins from m4MR-infected cell supernatant (lane 7) migrated slower than that from m3MR-infected samples (lane 5), indicating that glycosylation at N130 residue may be more complex and heterogeneous than glycosylation at residue N207. Furthermore, since NS1 was secreted in significantly fewer amounts from m3MR- and m5MR-infected cells (lanes 5, and 6, 9, and 10) compared to m2MR- or m4MR-infected cells (lanes 3 and 4, 7 and 8), it appears that glycosylation at N130 may be important for efficient secretion of NS1. However, a detailed quantitative analysis of secreted and intracellular levels of each of these proteins would be necessary to strengthen such a conclusion. 

### 3.3. m5MR Virus Encoding both Non-Glycosylated E and NS1 Proteins is Highly Attenuated

**Figure 3:**
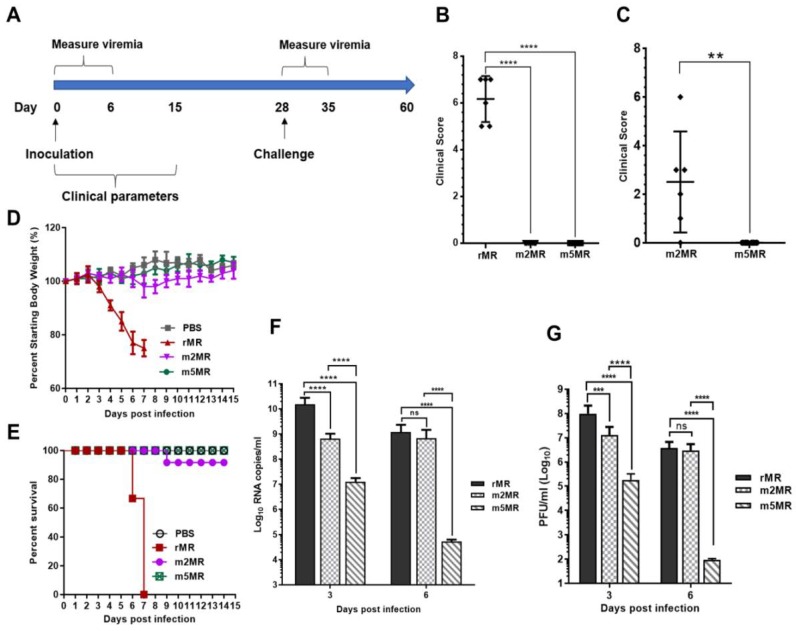
m5MR virus is attenuated in mice. (**A**) Scheme of virus inoculation and challenge study in three to four-week-old *Ifnar1*^−/−^ A129 mice. Mice were injected subcutaneously (s.c.) with 1000 PFU (plaque-forming unit) of rMR (*n* = 6), m2MR (*n* = 12), and m5MR (*n* = 12) viruses on day 0. PBS was injected in the control group (*n* = 6). Clinical scores at 6 dpi for rMR, m2MR, and m5MR virus-infected groups (**B**) and 10 dpi for m2MR and m5MR virus-infected groups (**C**). (**D**) Weight loss and (**E**) Percent (%) survival of mice in infected or PBS control groups. Viremia in serum at 3 dpi and 6 dpi as measured by genome copy numbers (**F**) and infectious virus titers (**G**). Data presented are combined data from two independent experiments. Unpaired Student’s *t*-test (two-tailed) (for panel (**B**,**C**)) and Mann–Whitney test (for panel (**F**,**G**)) were used to determine significance between groups. ****, *p* < 0.0001; ***, *p* < 0.001; **, *p* < 0.01; ns, non-significant.

To examine the effect of nonglycosylation of both the E and NS1 proteins on ZIKV pathogenicity in animals, three to four-week-old *Ifnar1*^−/−^ A129 mice were divided into four groups: mock (*n* = 8), rMR (*n* = 6), m2MR (*n* = 12), and m5MR (*n* = 12). Mice were inoculated subcutaneously (s.c.) with 1000 PFU of the virus except those in the mock group, which received an equal volume of PBS. Mice were monitored from 0 to 15 dpi for clinical scores, weight loss, and survival, and viremia in the first-week post-infection (Figure 3A). At 4–5 dpi, the rMR virus-infected mice started showing disease signs, such as shivering, conjunctivitis, and lethargy, as seen previously in our studies [49]. Subsequently, some mice showed both fore and hind limb paralysis, and by 6–7 dpi, all the mice developed severe disease signs and had to be euthanized. In contrast, mice in the m2MR and m5MR groups showed no symptoms at 6 dpi (Figure 3B). However, at 8–10 dpi, two to three mice from m2MR group showed mild paralysis, but most of them recovered by 11 dpi. Interestingly, all mice in the m5MR virus-infected group did not show any disease signs between 0–15 dpi (Figure 3C). The rMR virus-infected mice exhibited a weight loss of more than 20% by 6 dpi (Figure 3D) and were euthanized or died by 7 dpi (Figure 3E). Mice in the m2MR group showed mild weight loss, while those in the m5MR group and the PBS-inoculated control group exhibited no weight loss (Figure 3D). Only one animal in the m2MR infected group was moribund and, thus, euthanized (Figure 3E). Furthermore, survived mice from m2MR group or all mice from m5MR group did not show any weight loss, disease signs, or mortality until challenge at 28 dpi. Viral genome copy numbers at 3 dpi were highest in rMR virus-infected animals, whereas the m2MR and m5MR groups showed ~10–15-fold and ~1000-fold reductions, respectively, compared to mice in the rMR group (Figure 3F). However, at 6 dpi, rMR and m2MR infected animals showed a similar number of viral genome copies in blood. In contrast, the m5MR group showed significantly lower levels of viremia (Figure 3F). Infectious viral titers showed a similar trend as genome copies at 3 dpi and 6 dpi (Figure 3G). 

**Figure 4:**
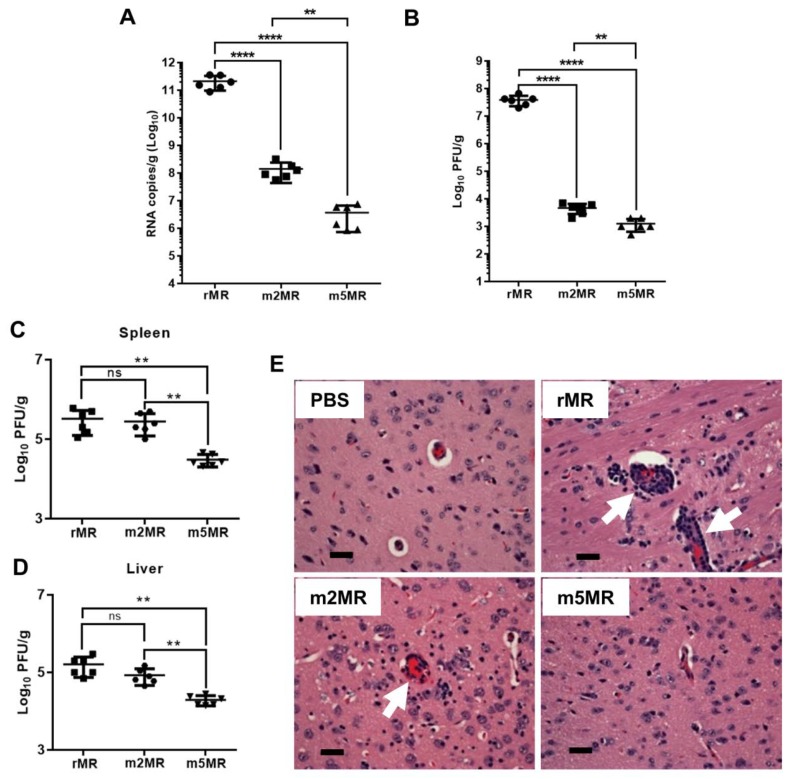
The mutant viruses replicate less efficiently in the brain of infected animals. Viral genome copy number (**A**) and infectious virus titer (**B**) in the brain of animals infected with rMR (*n* = 6), m2MR (*n* = 6), and m5MR (*n* = 6) viruses at 6 dpi. Infectious virus titers from the spleen (**C**) and liver (**D**) of the animals. Unpaired Student’s *t*-test (two-tailed) was used to determine significance between the groups. ****, *p* < 0.0001; **, *p* < 0.01. (**E**) Hematoxylin and eosin staining of sections of the cerebrum of animals inoculated with PBS, or infected with rMR, m2MR, or m5MR viruses. Representative images of the cerebrum (40x) from animals in each group are shown. Prominent perivascular cuffing in rMR- and m2MR-infected samples are shown with white arrows. Scale bar (at bottom left), 60 µm.

To measure viral replication and examine pathology in tissues, we sacrificed several mice from each group and collected tissues at 6 dpi. Viral genome copies in the brain of m2MR- and m5MR-infected mice were significantly lower compared to the rMR-infected mice (Figure 4A). The m5MR-infected mice had significantly lower genome copies (~90-fold less) as compared to those infected with m2MR virus (Figure 4A). Infectious viral titer in the brain followed a similar pattern between the groups (Figure 4B). Although replication of the m5MR virus in the brain was significantly restricted, the virus replicated to moderate levels in other tissues, such as spleen and liver. m5MR virus replicated six- to ten-fold less in these tissues compared to rMR and m2MR virus-infected mice (Figure 4C,D). Overall, these results indicate that m5MR virus replication is severely restricted in the brain and to some extent in the spleen and liver of the infected animals.

Histopathological examination of tissues corroborated the findings from the viral load. The brain sections of mice from the rMR-infected group showed generalized moderate to severe perivascular cuffing, which predominantly contained mononuclear cells with occasional neutrophils (Figure 4E). The meninges showed lymphocytic perivascular cuffing. Shrunken hyperchromatic neurons and occasional karyorrhectic cells were seen in the brains of rMR-infected mice. Brains in the m2MR group showed rare diffuse mild cuffing in the parenchyma along with occasional shrunken hyperchromatic neurons (Figure 4E). In contrast, perivascular cuffing in the brain parenchyma of m5MR-infected mice was rare (Figure 4E), while the meninges appeared normal, which was very similar to the brains of mice in the PBS injected group.

In rMR virus-infected mice, the spleen exhibited mild lymphoid hyperplasia with hypertrophied lymphocytes and occasional aggregates of neutrophils. Diffuse karyorrhexis was also noted in the spleen, whereas the liver showed few small mononuclear cell foci and prominent Kupffer cells, similar to our previous observations [49]. Overall, the pathological changes in the spleen and liver of m5MR-infected mice were minimal compared to those in the other virus-infected groups.

### 3.4. m5MR Virus Protects Immunocompromised Mice from Lethal Challenge

**Figure 5:**
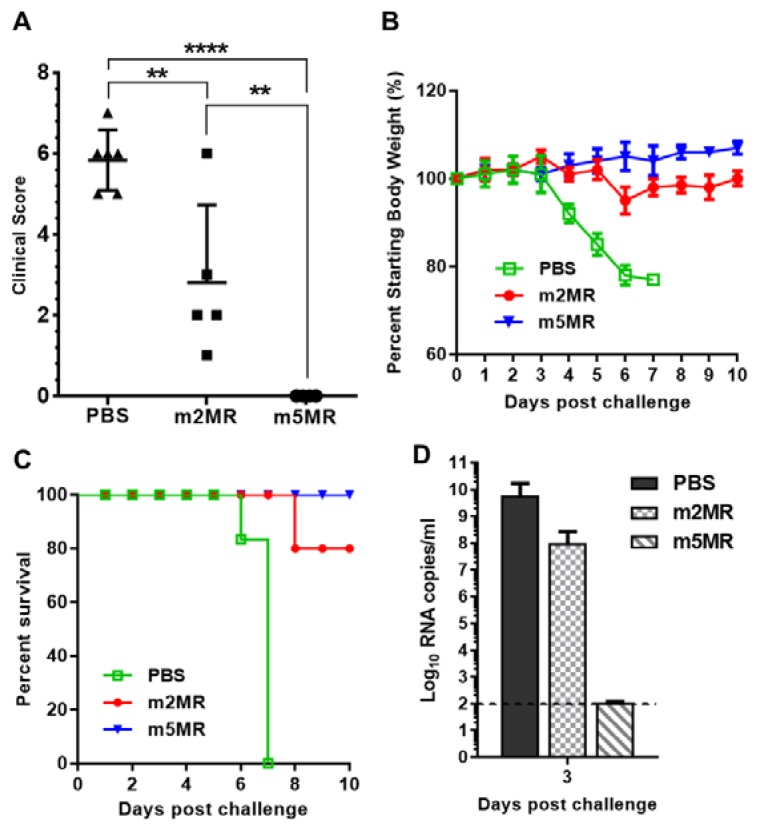
Mutant virus-infected animals are protected from lethal challenge. (**A**) Clinical scores, (**B**) weight loss, and (**C**) survival of the animals injected with PBS (*n* = 6), or infected with m2MR (*n* = 5), m5MR (*n* = 6), and after 28 days, challenged s.c. with 10,000 PFU rMR virus. (**D**) rMR viremia in the plasma following challenge. The horizontal dashed line shows the limit of detection. Unpaired Student’s *t*-test (two-tailed) for panel A was used to determine significance. ****, *p* < 0.0001; **, *p* < 0.01.

The majority of mice in the m2MR-infected group and all mice in the m5MR-infected group survived from the infection. Next, we challenged these surviving mice with a lethal dose of 10,000 PFU of rMR virus at 28 dpi to assess the level of protection conferred by m2MR and m5MR viruses. This high dose of virus was used as the mice were already 7–8 weeks old at this time of challenge, and it has been shown that ZIKV susceptibility in these mice is age-dependent [52,53]. The animals injected with PBS served as an age-matched control group. After the challenge, PBS-injected mice showed disease signs by 4 days post-challenge (dpc), and all the mice showed high clinical scores and succumbed to infection by 6–7 dpc (Figure 5A). In the m2MR-infected group, mice started showing mild disease signs, such as shivering and conjunctivitis on 7–8 dpc, and only one mouse succumbed to the infection on 8 dpc. No clinical signs were observed in mice infected with m5MR virus and challenged with rMR virus (Figure 5A). Mice in the PBS control group showed severe weight loss after challenge, whereas mild weight loss was observed in m2MR group and no weight loss in m5MR group (Figure 5B).

Interestingly, all mice in the m5MR group survived the challenge, whereas 80% of mice in the m2MR group and none in the PBS control group survived the challenge (Figure 5C). Additionally, we observed 2 log lower levels of challenge virus (rMR) viremia in m2MR-infected animals compared to PBS control group and undetectable levels of challenge virus (rMR) viremia in m5MR-infected animals following challenge (Figure 5D). Overall, the results show that the m5MR virus-infected animals were fully protected upon lethal virus challenge, whereas mice in m2MR group were partially protected compared to the m5MR group. These results suggest that protective immune responses were induced in these animals by the m5MR and m2MR viruses. 

### 3.5. Protection from Challenge is Conferred by both Humoral and Cell-Mediated Immune Responses

**Figure 6:**
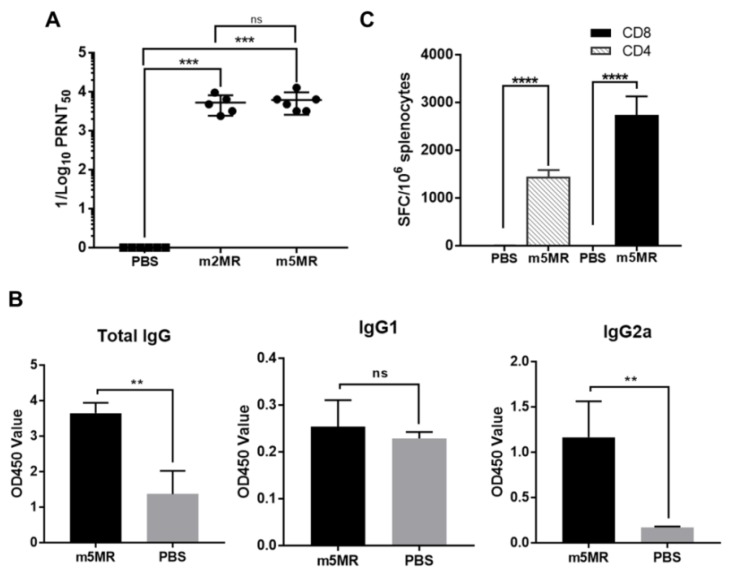
Humoral and cell-mediated immune responses in mutant virus-infected animals. (**A**) The viral neutralization titers in serum [expressed as reciprocal of 50% plaque reduction neutralization test (PRNT_50_) values] from PBS-treated or m2MR- or m5MR-infected animals at 28 dpi. (**B**) Antibody isotyping in sera by ELISA for PBS-injected or m5MR virus-infected mice. (**C**) The spleen from m5MR virus-infected (*n* = 5) or PBS-injected (*n* = 4) A129 animals were collected at 28 dpi, and the cells were stimulated with ZIKV E specific peptides for CD4+ and CD8+ T cells. ELISPOT was performed to determine spot-forming cells (SFC) per million splenocytes. Mann–Whitney test (for panel (**A**, **B**)) and Unpaired Student’s *t*-test (two-tailed) (for panel (**C**)) were used to determining significance between groups. ****, *p* < 0.0001; ***, *p* < 0.001; **, *p* < 0.01; ns, non-significant.

To understand the factors contributing to the protection observed in mutant virus-infected mice, serum was collected from animals in each group at 28 dpi, just before an rMR virus challenge, and neutralizing antibody titers were determined. Both m2MR and m5MR-infected animals had significantly high neutralization titers (PRNT_50_) with geometric mean antibody titer (GMT) of 4688 and 5434, respectively (Figure 6A). 

In a separate study, A129 mice were injected with PBS (*n* = 4) or m5MR (*n* = 5) virus, the sera and spleen were collected at 28 dpi. Examination of IgG isotypes in serum revealed that the m5MR group had significantly higher total IgG and IgG2a antibody responses as compared to those in the PBS control group (Figure 6B). The IgG1 was not significantly different in the mice between the two groups (Figure 6B).

We next examined the T cell response using IFN-γ ELISPOT assay. Splenocytes were harvested from mice and stimulated ex vivo with known peptide pools of 15-mer peptides (Table 2), spanning the entire envelope protein of ZIKV, which are capable of stimulating CD4+ and CD8+ T cell responses. The mice inoculated with m5MR virus exhibited strong CD4+ and CD8+ T cell immune responses as indicated by a significantly higher number of spot forming cells (SFC) per million cells compared to PBS group (Figure 6C), indicating induction of cell-mediated immune responses.

### 3.6. Passive Transfer of Antibodies from Animals Infected with m5MR Virus Protects Mice from Lethal Challenge

**Figure 7:**
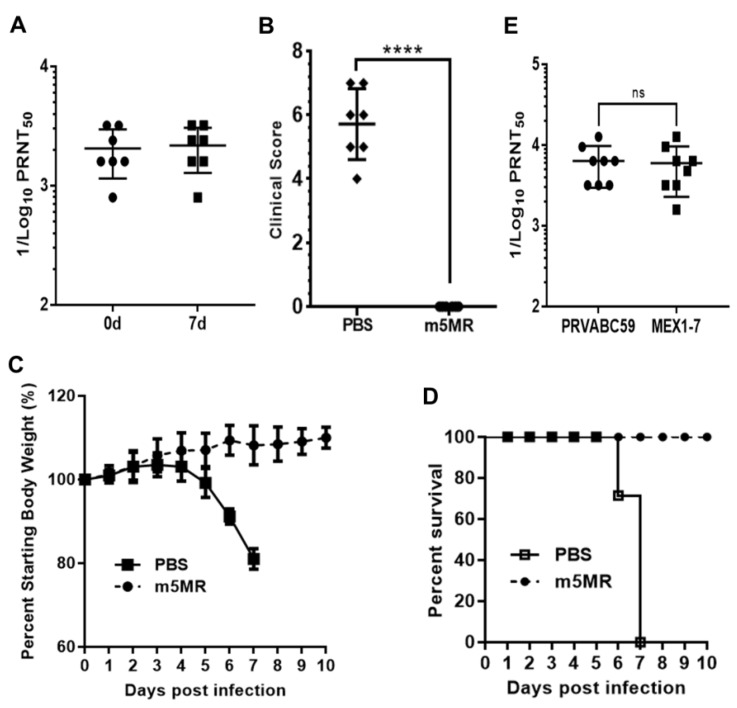
Passive transfer of sera from m5MR-infected animals protects naïve animals from lethal challenge. (**A**) The antibody neutralization titer following 2 hpt (0d) and 7-day pt (7d) of pooled sera in A129 mice. (**B**) Clinical scores, (**C**) weight loss, and (**D**) survival of animals challenged with a lethal dose of rMR virus following passive transfer of sera. PRNT_50_ titer of serum samples from individual animals infected with m5MR virus against PRVABC59 and MEX1–7 strains of ZIKV. The antibody titers are expressed as reciprocal of PRNT_50_ values. Unpaired Student’s *t*-test (two-tailed) for panel (**B**) and Mann–Whitney test for panel (**E**) were used to determine significance between the groups. ns, non-significant. hpt: hour post transfer of serum. ****, *p* < 0.0001; ns, non-significant.

To determine if the serum from m5MR virus-infected mice could confer protection against lethal rMR virus challenge, we performed passive antibody transfer experiment in A129 mice. One group of mice (*n* = 7) was inoculated s.c. with 200 μL of undiluted pooled sera from m5MR virus-infected A129 mice, while a control group of mice (*n* = 7) was administered with sera from naïve animals. Within 2-hour post-transfer (hpt), we collected sera from mice and verified the successful transfer by measuring the antibody neutralization titer. Indeed, the mice that received m5MR-infected sera showed neutralization with GMT of 1871 (Figure 7A), whereas mice inoculated with naïve sera showed no neutralization titers. After 2 hpt, the mice were challenged with 1000 PFU of rMR virus s.c. Following inoculation, mice in the control sera group showed severe weight loss, and all mice succumbed to infection by 6–7 dpc. In contrast, the mice injected with m5MR virus-infected immune sera showed no disease signs or weight loss, and all mice survived the challenge (Figure 7B–D). At the peak time of disease in control group of animals, i.e., on 7 dpc, we found high antibody neutralization titers (GMT: 1983) in the m5MR group as we had seen just before the challenge (Figure 7A). These results suggest that passive transfer of immune sera completely protected the mice against rMR virus lethal challenge. 

To examine if the antibodies, which were generated by the mutant MR766 virus, an African lineage virus, could neutralize the American/Asian lineage viruses isolated from recent outbreaks, we performed PRNT assay using the serum from m5MR virus-infected mice with PRVABC59 and MEX1–7 strains of ZIKV. Our results showed high neutralization titers against PRVABC59 (GMT: 6142) and MEX 1-7 (GMT: 5339) strains (Figure 7E), similar to that observed for MR766 strain (Figure 6A). These results suggest that m5MR virus immune sera may confer heterologous protection. 

## 4. Discussion

The recent emergence of ZIKV in the western hemisphere and the association of ZIKV with congenital malformations, such as microcephaly and Guillain-Barré syndrome, have raised a serious public health concern. To date, no clinically approved drugs or vaccines are available for ZIKV infection. Several vaccine platforms are being developed for ZIKV [54,55,56], and other flavivirus vaccine strategies are also being applied to ZIKV [57]. Platforms, such as inactivated and subunit vaccines, have shown to be effective in mice and nonhuman primate models [58,59,60] but none of them have been approved for clinical use as yet. Since live attenuated virus vaccines can often provide long-lasting protection with a single dose without the additional need for adjuvants or other carrier proteins, these platforms have significant advantages over subunit or killed vaccine strategies. Attenuation strategies through genetic manipulation of infectious clones have been employed for YFV, Japanese encephalitis virus (JEV), WNV, DENV-2, and DENV-4 virus, as well as ZIKV [39,42,55,61,62,63]. We and others had previously shown that a mutant ZIKV containing a single amino acid substitution (N154A) in the E protein glycosylation site was attenuated in pathogenicity and neuroinvasion [49,64,65]. Additionally, ZIKV encoding mutant NS1 protein without glycosylation has been shown to confer protection against fetal transmission [63]. Here, we attempted to further attenuate the pathogenicity of the virus by incorporating glycosylation site mutations in the NS1 protein in the genomic background of a virus with E glycosylation mutation (m2MR virus). Using the newly generated virus (m5MR) encoding non-glycosylated E and NS1 proteins, our results showed that the m5MR virus was stable, replicated less efficiently in cell culture, particularly in mosquito cells (C6/36) compared to the wildtype rMR virus, displayed complete attenuated phenotype in mice model, and exhibited mild to no pathology in tissues of infected animals. Also, m5MR virus protected animals from lethal challenge with rMR virus by eliciting strong humoral and cellular immune responses. Importantly, passive transfer of m5MR-infected mice sera completely protected the immunocompromised mice upon lethal rMR virus challenge. 

In our effort to determine the role of the two conserved NS1 glycosylation sites in replication and pathogenesis, we generated three mutant viruses by mutating the sites (N130 and N207) individually or together in the background of E protein glycosylation mutant virus. All the mutant viruses were found to be genetically stable under in vitro growth conditions as they retained the engineered mutations and revealed no additional nucleotide changes in the E and NS1 protein-coding sequences upon six passages in cell culture. On the other hand, such mutations in the NS1 glycosylation sites (N130 and N207) in DENV-2 led to virus instability, with multiple mutations in NS1 region arising after one passage in cells [45]. Whether mutant ZIKVs containing glycosylation mutations in both E and NS1 proteins are more stable than the DENV-2 mutant viruses carrying only the NS1 mutations or whether compensating mutations in other viral protein-coding regions are present in m5MR virus is not known at this time but could be investigated in the future. It should also be noted that unlike stable genotype of the m5MR virus mutant, a recent study described that PRVABC59 ZIKV and its infectious clone quickly accumulate stable mutations in E and NS1 proteins in mice, resulting in their attenuation of pathogenicity [66]. It is unclear at this time whether the sequence stability of our m5MR virus is related to the virus strain and the conditions of passage or that the PRVABC59 virus is more prone to sequence changes when passaged in mice. 

Examination of growth kinetics of the mutant viruses (m3MR, m4MR, and m5MR) in Vero and C6/36 cells revealed that glycosylation at each of the NS1 sites was required for efficient viral growth. Although m3MR and m4MR viruses grew to somewhat lower titers in these cells, the growth of m5MR virus was significantly inhibited. This is particularly true for m5MR virus growth in C6/36 mosquito cells in which the virus grew to significantly reduced titers. The localized foci of E-expressing cells during virus recovery after RNA transfection (Figure 1B) are also particularly noteworthy. Inefficient secretion of a nonglycosylated NS1 protein might be responsible for this phenotype. In this regard, it will be interesting to examine if replication and transmission of the m5MR virus are negatively impacted by mosquito vectors. 

Examination of the mobility differences between the NS1 proteins of m3MR and m4MR viruses suggested that N130 and N207 glycosylation sites might contain different glycan moieties. The NS1 of m3MR virus migrated slightly faster than that of the m4MR virus, indicating that sugar moiety at N130 might be larger and/or more complex than the moiety at N207. These results are consistent with previous observations from other closely related flaviviruses, showing that NS1 N130 has a complex glycan moiety and N207 contains high-mannose sugar moiety [29,32]. Although we had not conducted a thorough and quantitative determination of NS1 secretion and its glycosylation status, it appeared from our results, presented in Figure 2, that efficient secretion of NS1 required glycosylation at N130 residue whereas glycosylation at N207 did not have a significant impact on secretion. Our results with ZIKV are consistent with other related viruses, such as DENV, WNV, and YFV, showing that mutations in glycosylation sites of NS1 affect its hexamer stability and secretion [28,39,40,47,48]. 

The results presented here demonstrated the effects of NS1 glycosylation on ZIKV replication and pathogenesis. This was borne out from our observation of not only reduced growth and replication of the m5MR virus in cultured cells but also the absence of any disease signs or disease in susceptible mice. The difference in mean viral loads between m5MR and m2MR virus-infected mice in the brain (Figure 4A) suggested that ZIKV attenuation was influenced by the glycosylation status of the NS1 protein. It has been proposed that ZIKV NS1 may interact with the endothelial cells of the blood-brain barrier (BBB) and maternal/fetal interface, favoring viral entry into the brain and fetus, respectively [67]. It is possible that the nonglycosylated NS1 produced in m5MR-infected mice may not interact efficiently with endothelial cells. Additionally, the reduced rate of secretion of nonglycosylated NS1 may also contribute to the nonpathogenic property of the m5MR virus. Mild to no histopathological changes in the brain further strengthens our suggestion for the benign nature of the m5MR virus infection. 

Interestingly, the protection afforded by m5MR from rMR virus challenge is an important finding because the m5MR virus completely protected (100% survival) mice. Therefore, the m5MR virus could be potentially used as a live attenuated vaccine to induce protective immune responses against ZIKV. The increased levels of IgG2a antibodies in m5MR-infected mice indicate that Th1 type immune responses likely contribute to protection. Studies have shown that the Th1 type immune response stimulates IgG antibody subclasses that are important for mediating antibody dependent cellular cytotoxicity (ADCC) and complement activities in mice [20,68,69]. It is possible that upon rMR virus challenge, virus-infected cells present viral protein antigens on the cell surface, which are then targeted by IgG2a antibodies followed by recruitment of lymphoid cells bearing Fc-γ receptors, to kill infected cells by ADCC [19,20]. The importance of antibodies in protecting immunodeficient mice against WNV infection has been reported [70]. Since antibodies to NS1 protein of ZIKV and other flaviviruses are protective [4,19,23], it is also possible that the induction of antibodies against unglycosylated NS1 protein in m5MR virus-infected animals may be protective. 

Increased levels of CD8+ and CD4+ T cells in the m5MR-infected group are indicative of robust cellular immune responses. The strong CD8+ T cell response in m5MR-infected animals, most likely contributed to the clearance of virus from the blood and organs upon challenge, which is confirmed by lack of virus detection in plasma and tissues at 3 dpi and 6 dpi, respectively, after rMR virus challenge. Similar clearance of the virus from the brain after the intracranial challenge of the virus was earlier reported for ZIKV, and it was hypothesized that the CD8+ T cell response was the contributing factor for clearance [69]. Furthermore, it was shown previously that CD8+ T cells contribute to clearance of virus from tissues in WNV-infected mice by inducing death of the virus-infected cells, which express WNV epitopes with class I MHC molecules on the cell surface [71]. Our observations of strong neutralizing antibody response, as well as T cells responses, suggest that the antibody response, in addition to protecting mice from the initial virus infection, may have augmented viral clearance, while the cell-mediated immunity, particularly CD8+ T cells, may have been responsible for clearing the virus from the tissues by inducing virus-infected cell death. Indeed, it has been shown that both B and T cells are needed to restrict ZIKV infection in IFN-knockout immunocompromised mice [72]. Future studies involving the adoptive transfer of T cells to naïve animals and subsequent challenge experiments may reveal the extent of the role of T cells in protection. 

Given the high conservation of E and NS1 proteins among ZIKV isolates, the m5MR virus should be protective against most or all ZIKV strains and could potentially serve as a universal vaccine candidate. Although our protection studies did not include heterologous strains for the challenge, the results from in vitro antibody neutralization studies with other strains of Asian lineage viruses suggest that the protection afforded by m5MR virus could be extended to multiple ZIKV strains, as the antigenic structures among ZIKVs are similar [16,69]. For example, studies have shown that human convalescent sera equally neutralize different ZIKV strains [60]. Thus, it would be interesting to examine the protection conferred by m5MR virus to challenge with heterologous strains of ZIKV. With regard to m5MR virus as a vaccine candidate, the stability of passage 6 m5MR virus in Vero cells is significant, as Vero cells are the approved cell line for vaccine production with 5 days per passage [73,74], which is also similar with other live vaccine candidates for ZIKV [75].

## 5. Conclusions

Results presented here showed that a recombinant ZIKV (m5MR) encoding non-glycosylated E, as well as non-glycosylated NS1 proteins, exhibited a highly attenuated phenotype, induced higher levels of humoral and cell-mediated immune responses, and conferred complete protection against lethal challenge. Thus, m5MR virus could serve as a potential live viral vaccine candidate.

## Figures and Tables

**Table 1:** Primers used to generate non-structural protein 1 (NS1) glycosylation site mutants.

Primer and Name	Sequence (5’ to 3’) *	Application	Nucleotide Change
1. MR-701F	GTGTACGGAACCTGTCATC	Upstream primer to generate PCR product for cloning	
2. NS1-130-N to A-2864F	GCGGCAAAGACCGCCAACAGTTTTGTTGTC	N130A glycosylation mutation	AAC to GCC
3. NS1-207-N to A-3090F	GGATTGAAAGTGAAAAGGCTGACACATGGAG	Single (N207A) glycosylation mutation	AAT to GCT
4. NS1-207-N to A-3090R	CTCCATGTGTCAGCCTTTTCACTTTCAATCC	Double (N130A/N207A) glycosylation mutation	AAC to GCC and AAT to GCT in reverse
5. MR-3873R	GCCAGG**GCTAGC**AGCATGCTCTC**CCGCGG**TGTCCAATTGGC	Downstream primer to generate PCR product for cloning	

* Nucleotide sequences encoding the glycosylation sites are underlined. Sequences in bold-face represent NheI and SacII sites in primer MR-3873R.

**Table 2:** Peptides used for stimulation of T cells.

Peptide	Sequence *	Amino Acid Position in E (1–504)
1	IRCIGVSNRDFVEGM	1–15
2	IGVSNRDFVEGMSGG	4–18
3	SNRDFVEGMSGGTWV	7–21
6	SGGTWVDVVLEHGGC	16–30
8	DVVLEHGGCVTVMAQ	22–36
19	EVRSYCYEASISDMA	55–69
21	YEASISDMASDSRCP	61–75
24	SDSRCPTQGEAYLDK	70–84
25	RCPTQGEAYLDKQSD	73–87
102	SYSLCTAAFTFTKIP	304–318
103	LCTAAFTFTKIPAET	307–321
104	AAFTFTKIPAETLHG	310–324
119	VGRLITANPVITEST	355–369
120	LITANPVITESTENS	358–372
123	ESTENSKMMLELDPP	367–381
124	ENSKMMLELDPPFGD	370–384

* The peptide sequences specific to the ZIKV (Zika virus) envelope protein.
